# Supplementary material for: Dosing trajectories of antihypertensive agents among preterm neonates: A retrospective, cross-sectional analysis
Source: PLoS One. 2025 Dec 8;20(12):e0336994. doi: 10.1371/journal.pone.0336994 (PMC12685197; doi:10.1371/journal.pone.0336994)
Supplement: S1 File — (DOCX) [file pone.0336994.s001.docx]

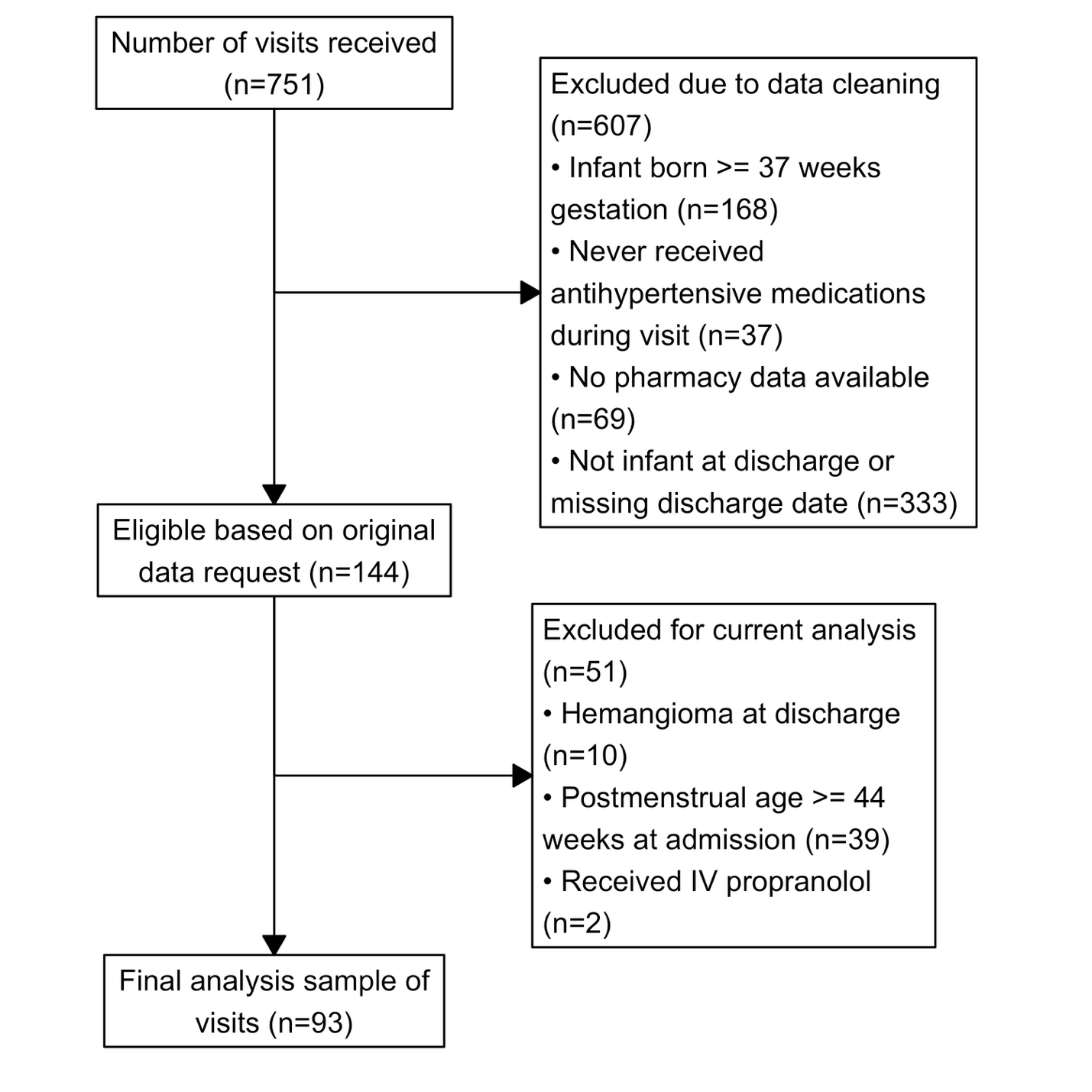


**Supplemental Figure 1.** Flow Diagram for inclusion and exclusion of patient visits.

**Supplemental Table 1.** **Prevalence of agents used to treat hypertension.** The denominator is all rounds of treatment with any of the target antihypertensive agents. If one visit had treatment with two different antihypertensive agents, both treatments were counted.

| **Characteristic** | **N = 114***^1^* |
| --- | --- |
| Antihypertensive Drug |  |
| Propranolol | 70 (61) |
| Esmolol | 14 (12) |
| Captopril | 10 (8.8) |
| Sodium nitroprusside | 6 (5.3) |
| Enalapril | 5 (4.4) |
| Hydralazine | 5 (4.4) |
| Clonidine | 4 (3.5) |
| *^1^*n (%) | |

**Supplemental Table 2. Baseline demographic and clinical characteristics for all patient visits, by whether diagnosis of neonatal hypertension was present.**

| **Characteristic** | **No diagnosis of neonatal hypertension N = 73*^1^*** | **Diagnosis of neonatal hypertension N = 9*^1^*** | **p-value*^2^*** |
| --- | --- | --- | --- |
| **Gender** |  |  | >0.9 |
| Female | 29 (40) | 3 (33) |  |
| Male | 43 (60) | 6 (67) |  |
| Unknown | 1 | 0 |  |
| **Race** |  |  | >0.9 |
| Asian | 1 (1.5) | 0 (0) |  |
| Black or African American | 28 (41) | 3 (38) |  |
| Hispanic or Latino | 0 (0) | 0 (0) |  |
| Other | 0 (0) | 0 (0) |  |
| White | 39 (57) | 5 (63) |  |
| Unknown | 5 | 1 |  |
| **Ethnicity** |  |  | 0.009 |
| Hispanic/Latino | 0 (0) | 2 (25) |  |
| Non-Hispanic/Latino | 70 (100) | 6 (75) |  |
| Unknown | 3 | 1 |  |
| **Gestational age at birth (weeks)** | 32.5 (29.2, 35.0) | 27.1 (25.0, 33.6) | 0.11 |
| **Birthweight (g)** | 1668 (897, 2354) | 864 (625, 2195) | 0.3 |
| Unknown | 35 | 6 |  |
| **Age at discharge (days)** | 40 (19, 75) | 91 (36, 99) | 0.2 |
| **Bronchopulmonary Dysplasia** | 20 (27) | 6 (67) | 0.026 |
| **Patent Ductus Arteriosus** | 26 (36) | 3 (33) | >0.9 |
| **Acute Kidney Injury** | 2 (2.7) | 1 (11) | 0.3 |
| **Congenital heart disease** | 40 (55) | 4 (44) | 0.7 |
| **Pulmonary hypertension** | 8 (11) | 0 (0) | 0.6 |
| *^1^*n (%); Median (Q1, Q3) | | | |
| *^2^*Fisher's exact test; Wilcoxon rank sum test; Wilcoxon rank sum exact test | | | |
