# Supplementary material for: Dosing trajectories of antihypertensive agents among preterm neonates: A retrospective, cross-sectional analysis
Source: PLoS One. 2025 Dec 8;20(12):e0336994. doi: 10.1371/journal.pone.0336994 (PMC12685197; doi:10.1371/journal.pone.0336994)
Supplement: Attachment — Submitted filename: Response to Reviewers.docx [file pone.0336994.s003.docx]

**Editorial comments**

***2. Thank you for stating the following financial disclosure:***
***We would like to acknowledge the University of Alabama at Birmingham Medical Center for providing the retrospective data used in this study. Research reported in this publication was supported by the National Center for Advancing Translational Sciences of the National Institutes of Health under award number UL1TR003096. The content is solely the responsibility of the authors and does not necessarily represent the official views of the National Institutes of Health.***

***Please state what role the funders took in the study. If the funders had no role, please state: "The funders had no role in study design, data collection and analysis, decision to publish, or preparation of the manuscript."***
***If this statement is not correct you must amend it as needed.***
***Please include this amended Role of Funder statement in your cover letter; we will change the online submission form on your behalf.***

Done

***3. Thank you for stating the following in the Acknowledgments Section of your manuscript:***
***We would like to acknowledge the University of Alabama at Birmingham Medical Center for providing the retrospective data used in this study. Research reported in this publication was supported by the National Center for Advancing Translational Sciences of the National Institutes of Health under award number UL1TR003096. The content is solely the responsibility of the authors and does not necessarily represent the official views of the National Institutes of Health.***

***We note that you have provided funding information that is not currently declared in your Funding Statement. However, funding information should not appear in the Acknowledgments section or other areas of your manuscript. We will only publish funding information present in the Funding Statement section of the online submission form.***
***Please remove any funding-related text from the manuscript and let us know how you would like to update your Funding Statement. Currently, your Funding Statement reads as follows:***
***We would like to acknowledge the University of Alabama at Birmingham Medical Center for providing the retrospective data used in this study. Research reported in this publication was supported by the National Center for Advancing Translational Sciences of the National Institutes of Health under award number UL1TR003096. The content is solely the responsibility of the authors and does not necessarily represent the official views of the National Institutes of Health.***

***Please include your amended statements within your cover letter; we will change the online submission form on your behalf.***

We have removed the funding statement from the Manuscript and included our updated funding statement in our Cover Letter.

**4. *In the online submission form, you indicated that the study authors are prevented from sharing the data publicly due to restrictions placed upon use of the data by the data owners. To initiate a request for the dataset, please contact*** [***ResearchData@uabmc.edu***](mailto:ResearchData@uabmc.edu) ***and reference request “i2b2.lds.inpatient infants receiving antihypertensives w/dx codes <37 pt set”. The corresponding author (***[***matthew_loop@auburn.edu***](mailto:matthew_loop@auburn.edu)***) will facilitate obtainment of the data for reasonable requests.***

***All PLOS journals now require all data underlying the findings described in their manuscript to be freely available to other researchers, either a. In a public repository, b. Within the manuscript itself, or c. Uploaded as supplementary information.***
***This policy applies to all data except where public deposition would breach compliance with the protocol approved by your research ethics board. If your data cannot be made publicly available for ethical or legal reasons (e.g., public availability would compromise patient privacy), please explain your reasons on resubmission and your exemption request will be escalated for approval.***

Thank you. We have included the following text in the appropriate box on the resubmission.

“These data cannot be shared publicly for legal reasons. The data use agreement signed between the authors and the University of Alabama at Birmingham prevents the authors from sharing the data publicly.”

***5. Please upload a copy of Figure 4, to which you refer in your text on page 11. If the figure is no longer to be included as part of the submission please remove all reference to it within the text.***

We apologize for this oversight. We have removed reference to Figure 4 from the text.

***6. Please include captions for your Supporting Information files at the end of your manuscript, and update any in-text citations to match accordingly. Please see our Supporting Information guidelines for more information:*** [***http://journals.plos.org/plosone/s/supporting-information***](http://journals.plos.org/plosone/s/supporting-information)***.***

Done

***7. If the reviewer comments include a recommendation to cite specific previously published works, please review and evaluate these publications to determine whether they are relevant and should be cited. There is no requirement to cite these works unless the editor has indicated otherwise.***

Thank you for your detailed editorial comments.

**Reviewer 1**

***Thank you for the opportunity to review this timely and clinically relevant manuscript titled:***
***“Dosing trajectories of antihypertensive agents among preterm infants: A retrospective, cross-sectional analysis.”***

***The study addresses an important gap in neonatal pharmacotherapy by characterizing real-world dosing patterns for antihypertensive agents in a preterm population. The authors appropriately apply functional K-means clustering to time-based dosing data, and the manuscript is generally well-written and well-structured.***

We thank the reviewers for their kind and encouraging comments.

***However, several clarifications and improvements are needed to enhance the interpretability and generalizability of the findings.***

***Minor Revisions***
***1. Justification for study population criteria (Page 3, Lines 59–60; Page5, Lines 122-123 ) Please explain why infants with a gestational age <37 weeks and a postmenstrual age <44 weeks were selected. While these thresholds are reasonable, a brief justification would enhance clarity for readers less familiar with NICU conventions.***

We thank the reviewer for recommending we provide a little more detail on why these cutoffs are important for non-NICU familiar readers. We have added text to the beginning of the Methods section, and we reproduce it below for the reviewer’s convenience:

“Our ideal target population was preterm (born < 37 weeks gestation) neonates (< 44 weeks postmenstrual age) treated for hypertension. Postmenstrual age is the age of the infant since the mother’s last menstrual period, and it includes both time spent in the womb (gestational age) plus time outside the womb (i.e., postnatal age). We chose this population because preterm infants have higher risk for hypertension,[21,22] and hypertension often resolves as infant age increases.[23]”

***2. Limitations of cross-sectional design (Page 3, Line 59; Page 5, Line 120; Page 18, Lines 327–335) As the study is cross-sectional, it does not assess outcomes such as blood pressure control or adverse effects. This limits the ability to draw clinical conclusions about dosing effectiveness. Please acknowledge this more explicitly in the Discussion section. Suggested addition: “Because this study does not follow patients longitudinally, it cannot evaluate therapeutic outcomes or causal effects. Future cohort studies are needed to assess the effectiveness and safety of specific dosing patterns.”***

We thank the reviewer and agree that we should have made this limitation more explicit. We have included the following text at the beginning of the “Limitations” paragraph:

“Because this study did not follow patients longitudinally, it could not evaluate therapeutic outcomes of these neonates. Future cohort studies are needed to assess the effectiveness and safety of these different dosing patterns.”

***3. Small sample sizes in some clusters (Page 13–14) Captopril and esmolol clusters include very small subgroups (e.g., n=2). Please add a cautionary note that these subgroup findings may not be generalizable.***

We agree and thank the reviewer for this comment. In light of this reviewer’s comment, as well as other reviewers, we have decided to implement null hypothesis significance testing for comparisons among clusters. Therefore, we have removed any statements describing variations across clusters in our *sample* that did not result in statistically significant inference. Therefore, we believe adding the cautionary note is no longer needed.
***4. Define "Dosing Trajectory" (Title) Consider briefly defining “dosing trajectory” early in the Introduction for clarity (e.g., “a patient’s pattern of dose administration over time”). Many readers are not familiar with this terminology.***

We thank the reviewer for this wise suggestion. The first sentence of the second paragraph of the Introduction now reads as follows:

“Treating hypertension with a specific pattern of dose administration over time (i.e. a “dose trajectory”) in preterm infants is challenging because there are no treatment guidelines or formal statements from the American Academy of Pediatrics, American College of Cardiology, or American Heart Association.”

***5. Language and Clarity Minor grammatical issues are present. Examples:***
***• Page 4, Line 84: “...hypertension problematic to identify...” → “...making hypertension difficult to identify...” A brief language review would improve readability. But this is just my suggestions.***

We thank the reviewer for this comment. We have addressed this specific comment, as well as performed additional language review to improve readability.

***6. Figure S1 is not included in the text (pdf file). (Page 8; Line 188) If possible, please provide it to me later.***

We apologize that the reviewer was not able to review the Figure S1. We have included it here below for the reviewer’s convenience.

**Supplemental Figure 1.** Flow Diagram for inclusion and exclusion of patient visits.

***This is a valuable, data-rich study that provides important descriptive insights into antihypertensive prescribing in neonates. With minor revisions, it will make a strong contribution to neonatal literature.***

We again thank the reviewer for these encouraging comments, and we agree that this manuscript could be a strong contribution to the neonatal literature, especially after including the reviewer’s recommendations.

**Reviewer 2**

***Firstly, thank you for this interesting look at data in a very understudied population. This data involves a degree of conjecture around modeling but it is an absolutely vital first step in getting to drug dosing that better balances safety and efficacy and lays the groundwork for more controlled studies. It can significantly inform day-to-day clinical practice (including my own). The quality of this manuscript overall is very good. Thank you!***

We wish to sincerely thank the reviewer for their kind words. We agree that this manuscript involves some conjecture, but that research has to start somewhere. We are glad that the reviewer found the manuscript helpful.

***Introduction:***
***I really don’t have a lot to comment on in this section. Overall, I believe it is sufficient in terms of describing the landscape and is well written. Normally I would ask for the authors to speak to existing literature—however, for this particular topic, much of what is available is very old or consensus/editorial rather than robust analysis. I appreciate the insight into publication rates and the issues with data in this population.***

***The biggest question I had was the choice of agents to analyze—specifically captopril, propranolol, and esmolol. The methods may be a better place for this information but I did not see it discussed there either. Were the top 3 just allocated for k means analysis arbitrarily? Was there a percentage cutoff for which would be included? Is there any description of what other drugs were used and their prevalence?***

We thank the reviewer for their question. We have now included a table of prevalences of all the target drugs observed in our study sample in the supplemental material as Supplemental Table 1. We regret not including this table in the first submission as supplemental material. We include the table below for the reviewer’s convenience:

| **Characteristic** | **N = 114***^1^* |
| --- | --- |
| Antihypertensive Drug |  |
| Propranolol | 70 (61%) |
| Esmolol | 14 (12%) |
| Captopril | 10 (8.8%) |
| Sodium nitroprusside | 6 (5.3%) |
| Enalapril | 5 (4.4%) |
| Hydralazine | 5 (4.4%) |
| Clonidine | 4 (3.5%) |
| *^1^*n (%) | |

***Was there a conscious choice to exclude diuretics? Indication overlap (most notable with BPD)? Better established dosing? Was usage too small? These are not mentioned at all in the list of drugs cross referenced. While not likely to be the most efficacious drugs their use in clinical practice for mild hypertension in neonates is definitely common, particularly loops and thiazides. I think at least addressing these drug classes and any exclusion rationale is warranted. Amlodipine is another that comes to mind, but that was included in the list of drugs used to identify potential patients to include and perhaps just didn’t have enough usage to be included?***

We thank the reviewer for their detailed thoughts and comments about the selection of agents to look for. To begin with, we looked for the following agents: vasodilators (hydralazine, nitroprusside, clonidine), angiotensin-converting enzyme (ACE) inhibitors (enalapril, captopril), beta-blockers (propranolol, labetalol, esmolol, atenolol, metoprolol), and calcium channel blockers (amlodipine, isradipine, nifedipine, nicardipine). Any agents that did not appear in the prevalence table above were not identified in our analysis sample (e.g., amlodipine). Follow-up with the University of Alabama at Birmingham confirmed that atenolol and isradipine are non-formulary at that institution for this patient population.

We identified these medications based on the medications identified in a large, multi-site study of antihypertensive drug exposure in NICUs using the Pediatrix database (Ravisankar et al. 2017), as well as additional team input. According to the Ravisankar et al (2017) manuscript, they excluded diuretics “due to their frequent use for other indications (such as, prevention and treatment of bronchopulmonary dysplasia)” (p. 3). As our manuscript was a first attempt to identify dosing trajectories, we decided remain consistent with prior literature on excluding diuretics. Additionally, because our case identification was based on the medication received, it was critical to try and avoid including cases where a particular drug was used for a non-hypertensive indication. Therefore, the exclusion of diuretics helped us to exclude cases where the drug was used for bronchopulmonary dysplasia and not hypertension, at the cost of having a smaller sample size for our study.

To make this exclusion of diuretics more clear for the reader, we have included the follow text in the Methods section, right after the list of drugs we are targeting:

“We did not include diuretics in order to be consistent with prior literature on antihypertensive drug exposure,[24] since including diuretics would have likely identified many cases of bronchopulmonary dysplasia.”

Ravisankar S, Kuehn D, Clark RH, Greenberg RG, Smith PB, Hornik CP. Antihypertensive drug exposure in premature infants from 1997 to 2013. *Cardiol Young*. 2017;27: 905–911.


***Methods:***
***125-128: what was the exclusion of IV propranolol for? I see it on the supplemental materials as well and it just sticks out as there weren’t other very specific exclusions like this. Large difference between PO and IV dosing or the short timeframes of administration? Though a broad exclusion, I would concur with the authors on excluding hemangioma—even if it is possible that patients might have coincidentally had propranolol received for hypertension and a hemangioma diagnosis code without pharmacologic treatment of the hemangioma…would be far too confounding to leave in.***

We thank the reviewer for their comment. IV propranolol was excluded because only 2 patients received propranolol via this route. Because oral propranolol is not highly bioavailable (~25%), oral and IV doses are not equivalent and thus cannot be directly compared. Therefore, it would be problematic to treat the dose trajectories of IV and oral propranolol similarly in the clustering analysis, since the propranolol exposures would be different between those receiving oral and IV doses.

We have updated the Methods section to clarify this exclusion, and we provide the updated text below for the reviewer’s convenience:

“Patients were excluded if they received IV propranolol because oral propranolol, the dominant route, has a bioavailability of approximately 25%. Therefore, oral and IV dose trajectories do not result in equivalent exposure to propranolol and could not be treated as equivalent in the clustering analysis. Only two patients received IV propranolol.”

***133-134: While I get what the gist was, the statement “the primary outcome of this study was the weight-based dose of antihypertensive…” is not technically correct. I think the outcome here is the characterization of doses administered, whether it be trajectory, distribution, etc. If one is calling something an outcome, I think the description of that needs to be technically correct and very narrow, though others may disagree with me here.***

We thank the reviewer for the comment. We have changed the first sentence of the “Outcome” subsection of the Methods to state:

“The primary outcome of this study was the weight-based dosing trajectory of the antihypertensive agent administered.”

***135-139: The dose standardization methodology and also addressing weight appears to be appropriate*.**

We appreciate the reviewer’s agreement with our approach.

***140-142: Just to be clear—if no additional doses were given in 6 hours would the data point for dosing trajectory purposes cease there? With as many of these agents that are dosed far less frequently than every 6 hours, I’m trying to understand how this impacts the flow of the data. If a patient is getting a medication every 12 hours, for example, then each dose would be its own point and not be carried forward?***

We thank the reviewer for the comment, and we sincerely apologize for the confusion our description created. The reviewer’s understanding is incorrect, and we will try to improve our description of what we did, both here and in the manuscript text.

Every participant had a value for the dose of antihypertensive drug they were on for every hour they were on the drug. The value for each hour was equal to the most recent dose until they received a new administration of the drug X hours later. X could be any number of hours.

The setting of the dose to be 0 occurred at 6 hours after the last dose they received. We implemented this rule so that participants who were on the drug for a short amount of time would not be kept at that final dose throughout the duration of the analysis. For example, the dose trajectory analysis for propranolol included a window of 1,000 hours. If a participant was on the drug for only 50 hours, and the final dose they received was 2 mg/kg, we did not want to keep their dose at 2 mg/kg from hours 51 to 1,000 for the clustering analysis. That would not accurately reflect the treatment regimen they were under from hour 51 onwards (i.e., “no treatment”). Thus, at hour 56, we changed their dose from 2 mg/kg to 0 mg/kg, in order to accurately reflect that they were no longer receiving the drug.

We have changed the text in the “Outcome” subsection of the Methods section to the following:

“To describe the dosing trajectories, the doses of oral medications were set to be constant for each hour until an additional dose was given or a dose change was made. After the last dose received by a given patient during follow up was administered, we kept that dose constant until six hours after the final administration of the drug, at which point the dose was set to be 0 mg/kg, reflecting the end of the treatment course. An interval of six hours was chosen because this was the most frequent dosing interval, especially for propranolol which was the most prevalent medication.”

***155-165: This explanation of the follow-up time makes sense. Thank you for including.***

We appreciate the reviewer’s comment.

***179-185: This might be an editorial question, but does this information concerning data requests need to be included in the text of the manuscript?***

We thank the reviewer for the question. Such statements are required by PLoS One, and we have included such statements in a previous paper we have published in PLoS One using the same dataset (Perez et al., 2025).

Perez AD, Pittman M-C, Afroz Tanni K, Sanderson KR, Park J, Feig DI, et al. Sequence of antihypertensive medications used in preterm infants with hypertension: A cross-sectional study. PLoS One. 2025 Apr 22;20(4):e0320002.

***Results:***
***For all tables, it would be helpful to remove units, including percents, from the numbers reported…for example table 1 might be “ethnicity, n (%)” or as you have done using the footnote to denote units. In that case, they don’t need to be included again in the data reported and it can just be “84 (98)” or similar. The specificity of rounding and sig figs seems to vary—can this be streamlined? Also, some clarification on what is being reported re: formatting. For birthweight in particular, the use of commas in confusing as to what numbers I’m looking at. The IQR is 897 to 2400 or are you reporting 897, 2, and 2400?***

We thank the reviewer for their observation and made the recommended changes to the tables.

***“Percent of invasive SBPs above the 90th percentile up to 1 day prior to the first drug administration (%) based on 1987 task force” is so long to include as an item in a table—can this be simplified and the description be noted as a superscript and then a footnote under the table? I also question how much these SBP endpoints actually add to the descriptive analysis here. I can understand why it might be included, but in the context of this study it seems to be a little extraneous, especially given there is a decent amount of missing data for this. Could it be summarized in the text to characterize the clinical appropriateness of therapy without needing to include so much detail?***

After considering the reviewer’s position, we have chosen to remove these statistics from the tables. We hope that this change makes the tables much easier to follow for the reader.

***Table 2: Again, the items in the table are kind of lengthy, can you specify the dosing units for the different drugs in a footnote instead of repeating them in each row?***

We thank the reviewer for this comment. We have implemented the proposed change.

***236-242: Concise. Great! I would avoid using dashes for ranges and use the actual word “to” to clean up numbers in text.***

We thank the reviewer for their comment and have made this revision to the text.

***Discussion:***
***Largely OK.***

***Line 318 where you start talking about strengths of the study comes off oddly to me, as most studies don’t explain why their work is strong and rather state the strengths in context of the discussion…I don’t if other reviewers would agree, but I would consider reworking that. I do appreciate the detail given to limitations as well!***

We thank the reviewer for their comments. As this practice is common in the Corresponding Author’s field, we will leave this change up to the editor to decide whether to include a section mentioning the study strengths. We are happy to remove the section.

**Reviewer 3**

***Methods***

***1- Page 5, Line 128. No clear rationale is provided for excluding IV propranolol if it was used for hypertension, potentially biasing the study by omitting a relevant treatment modality. The authors must explicitly justify this exclusion.***

We thank the reviewer for their comment. IV propranolol was excluded because only 2 patients received propranolol via this route. Because oral propranolol is not highly bioavailable (~25%), oral and IV doses are not equivalent and thus cannot be directly compared. Therefore, it would be problematic to treat the dose trajectories of IV and oral propranolol similarly in the clustering analysis, since the propranolol exposures would be different between those receiving oral and IV doses.

We have updated the Methods section to clarify this exclusion, and we provide the updated text below for the reviewer’s convenience:

“Patients were excluded if they received IV propranolol because oral propranolol, the dominant route, has a bioavailability of approximately 25%. Therefore, oral and IV dose trajectories do not result in equivalent exposure to propranolol and could not be treated as equivalent in the clustering analysis. Only two patients received IV propranolol.”

***2- Page 6, Line 135-140. Treating IV boluses and infusions identically as mg/kg/minute overlooks distinct pharmacokinetic profiles and clinical strategies, potentially obscuring important nuances in dosing trajectories. The authors must justify this methodological choice***

After reviewing the data, we confirmed that no IV boluses were given to any of the patients on esmolol (i.e., they received continuous infusions). Therefore, we have removed the statement from the methods section stating that we did not distinguish between IV infusions and boluses. We thank the reviewer for the comment, which helped us to clarify to the reader which types of administrations were present in our dataset.

***3- Page 7, Lines 164-165. The ad hoc visual inspection was used to choose follow-up cutoffs (1,000 hours for propranolol, 400 hours for captopril, and 250 hours for esmolol). The authors must provide a rationale for these cutoffs***

We thank the reviewer for their careful critique. After considering this critique, we decided to implement a more objective approach to deciding on the window of hours to consider for each drug. Because we were trying to choose a window that maximized the variability in the curve trajectories, we decided to go with the following approach. First, we considered a span of different possible windows. At each possible window, we then computed the pairwise distances between each curve. Second, we calculated the standard deviation of those distances. Finally, we chose the window that *maximized* the variance of those distances. (For esmolol, there appeared to be an unrealistically short time frame at which maximum variance was reached (1 hour), so we chose an approximate local maximum at 50 hours in order to increase the number of doses to inform the clustering for esmolol.)

The window that maximized the differences between the curves was 868.3 hours for propranolol, 336.83 hours for captopril, and 50 hours for esmolol. All of the clustering analyses have been updated with these new windows, and we think this change has made the paper more objective. We have also updated the Methods section to describe this approach, and we reproduce the text below for the reviewer’s convenience:

“Therefore, we used the following approach. First, we considered a span of different possible windows. At each possible window, we then computed the pairwise distances between each curve. Second, we calculated the standard deviation of those distances. Finally, we chose the window that maximized the standard deviation of those distances for each drug. For esmolol, there appeared to be an unrealistically short time frame at which maximum variance was reached (1 hour), so we chose an approximate local maximum at 50 hours to increase the number of observations for esmolol dose trajectories. The cutoffs for the time windows were 868.3 hours for propranolol, 336.83 hours for captopril, and 50 hours for esmolol.”

***4- The manuscript lacks objective criteria or statistical measures for selecting the optimal number of clusters for each drug***

We thank the reviewer for this comment. The challenge with deciding on an optimal number of clusters for our dataset is that the low sample sizes create large uncertainty for the current methods for choosing the optimal number of clusters. In the table below, we show that the optimal number of clusters identified via 10 different methods ranged from 2 to 6 clusters for each of the drugs. While the results may be more reliable for propranolol, which has a larger sample size, creating 6 clusters out of 10 curves (captopril) or 14 curves (esmolol) seems unwise. Therefore, respectfully, we would like to keep our original approach of showing results across a range of numbers of possible clusters (2 to 4). We hope that this response satisfies the reviewer’s concern.

| drug | Calinski.Harabatz | Calinski.Harabatz2 | Calinski.Harabatz3 | Ray.Turi | Davies.Bouldin | BIC | BIC2 | AIC | AICc | AICc2 |
| --- | --- | --- | --- | --- | --- | --- | --- | --- | --- | --- |
| Propranolol | 2 | 6 | 4 | 3 | 4 | 2 | 2 | 2 | 2 | 2 |
| Captopril | 6 | 6 | 6 | 2 | 3 | 2 | 2 | 2 | 2 | 2 |
| Esmolol | 2 | 6 | 6 | 3 | 3 | 2 | 6 | 2 | 2 | 2 |

***Results***
***1- Pages 13, 15; Tables 3b, 3c. The small sample sizes in some clusters for captopril (N=2, N=3) and esmolol (N=2, N=3) severely limit the reliability and interpretability of findings for these drugs.***

We agree and thank the reviewer for this comment. In light of this reviewer’s comment, as well as other reviewers, we have decided to implement null hypothesis significance testing for comparisons among clusters. Therefore, we have removed any statements describing variations across clusters in our *sample* that did not result in statistically significant inference. Therefore, we believe adding the cautionary note is no longer needed.

***2- Pages 13, 15. The high percentage of Unknown values for critical variables such as birthweight in the cluster analyses is problematic, especially when birthweight is highlighted as a differentiating factor. The authors must discuss the impact of this missing data on validity and generalizability***

We thank the reviewer for this critique. We have now added text highlighting the large proportion of missing data for birthweight to both the Results and Discussion section. We reproduce that new text below for the reviewer’s convenience.

Results: “A large proportion of participants were missing birthweight (55%).”

Discussion: “The high proportion of patients missing birthweight when querying their electronic medical records using the UAB clinical data warehouse likely prevented detection of statistically significant differences by birthweight across some clusters.”

***3- Page 12, line 237. initial doses ff 0.1−0.2 mg/kg. "ff" Should be "of".***

Done

***Discussion***
***1- Only 10% of patients had a formal hypertension diagnosis despite all receiving antihypertensives. This significant discrepancy between medication use and formal diagnosis is underexplored.***

We agree with the reviewer that we would have preferred if a higher percentage of patients in our sample had a formal discharge diagnosis of neonatal hypertension in their records. We obtained the diagnosis of neonatal hypertension from discharge ICD codes and code descriptions. Other studies such as Ravisankar *et al*. (2017) were able to review notes in the patient records and determine whether the attending physician ever mentioned high BP. In that manuscript, which focused on infants born <= 32 weeks and <= 1500 g with no congenital anomalies, the positive predictive value of a diagnosis of hypertension, given that a patient was treated with one of the agents we focused on, was 75%.

Ravisankar *et al*. (2017) had access to more details about their participants than we did, so it is expected that they found more evidence of neonatal hypertension than we did, as we relied solely on discharge diagnosis codes.

Because inpatient pharmacy data is likely collected with more fidelity and consistency across studies than discharge diagnosis codes, and both our study and the Ravisankar et al. (2017) study used the same set of antihypertensive drugs to identify treated patients, we think our patient set is likely to have closer to 75% patients with hypertension than the 10% estimated in our study with a more error-prone measurement of detecting neonatal hypertension (i.e., discharge diagnosis codes).

We regret that we did not make this clearer in the previous version of the manuscript. We have added the following text to the Methods section:

“We used receipt of an antihypertensive medication as a proxy for receipt of treatment for hypertension. A previous study of a patient population born <= 32 weeks gestational age and <= 1500 g with no congenital anomalies found that receipt of an antihypertensive medication had a positive predictive value of 75% for having a mention of hypertension in the medical record.[24]”.

We have also added the following text to the Discussion section:

“Additionally, a prior study of over 100,000 NICU admissions found that receipt of any of these medications had a positive predictive value of 75% for a diagnosis of hypertension.[24]”

Also, in an attempt to explore possible differences between visits with a diagnosis of neonatal hypertension versus no diagnosis of neonatal hypertension as the reviewer recommended, we have created Supplemental Table 2, which we reproduce below for the reviewer’s convenience. Assuming a false positive rate of 0.05, the only differences we could detect statistically were a higher rate of visits among Hispanic/Latino patients among those with a hypertension diagnosis (25%) versus without a diagnosis (0%), as well as a higher prevalence of discharge diagnosis codes for bronchopulmonary dysplasia among visits with a diagnosis of neonatal hypertension (67%) versus no diagnosis (27%). Given that bronchopulmonary dysplasia is one known cause of neonatal hypertension, this finding is not surprising.

**Supplemental Table 2. Baseline demographic and clinical characteristics for all patient visits, by whether diagnosis of neonatal hypertension was present.**

| **Characteristic** | **No diagnosis of neonatal hypertension N = 73*^1^*** | **Diagnosis of neonatal hypertension N = 9*^1^*** | **p-value*^2^*** |
| --- | --- | --- | --- |
| **Gender** |  |  | >0.9 |
| Female | 29 (40%) | 3 (33%) |  |
| Male | 43 (60%) | 6 (67%) |  |
| Unknown | 1 | 0 |  |
| **Race** |  |  | >0.9 |
| Asian | 1 (1.5%) | 0 (0%) |  |
| Black or African American | 28 (41%) | 3 (38%) |  |
| Hispanic or Latino | 0 (0%) | 0 (0%) |  |
| Other | 0 (0%) | 0 (0%) |  |
| White | 39 (57%) | 5 (63%) |  |
| Unknown | 5 | 1 |  |
| **Ethnicity** |  |  | 0.009 |
| Hispanic/Latino | 0 (0%) | 2 (25%) |  |
| Non-Hispanic/Latino | 70 (100%) | 6 (75%) |  |
| Unknown | 3 | 1 |  |
| **Gestational age at birth (weeks)** | 32.5 (29.2, 35.0) | 27.1 (25.0, 33.6) | 0.11 |
| **Birthweight (g)** | 1,668 (897, 2,354) | 864 (625, 2,195) | 0.3 |
| Unknown | 35 | 6 |  |
| **Age at discharge (days)** | 40 (19, 75) | 91 (36, 99) | 0.2 |
| **Bronchopulmonary Dysplasia** | 20 (27%) | 6 (67%) | 0.026 |
| **Patent Ductus Arteriosis** | 26 (36%) | 3 (33%) | >0.9 |
| **Acute Kidney Injury** | 2 (2.7%) | 1 (11%) | 0.3 |
| **Congenital heart disease** | 40 (55%) | 4 (44%) | 0.7 |
| **Pulmonary hypertension** | 8 (11%) | 0 (0%) | 0.6 |
| *^1^*n (%); Median (Q1, Q3) | | | |
| *^2^*Fisher's exact test; Wilcoxon rank sum test; Wilcoxon rank sum exact test | | | |

We have included the following additional text in the Results section:

“Table S2 shows the same demographics analysis as Table 1, but by presence of a diagnosis for neonatal hypertension. Assuming a false positive rate of 0.05, the only differences we could detect statistically were a higher rate of visits among Hispanic/Latino patients among those with a hypertension diagnosis (25%) versus without a diagnosis (0%), as well as a higher prevalence of discharge diagnosis codes for bronchopulmonary dysplasia among visits with a diagnosis of neonatal hypertension (67%) versus no diagnosis (27%).”

**Reviewer 4**

***1. General Assessment*
*The manuscript addresses a relevant and under-explored topic in the literature. The authors’ work represents a meaningful contribution to the field and has the potential to advance understanding in this area. However, several important methodological and presentation-related issues should be addressed to improve the clarity and rigor of the study.*

*2. Clarification of Inclusion Criteria*
*The manuscript frequently refers to the “treatment of hypertension” in the studied cohort. However, based on the inclusion criteria described, it appears that patients were selected based on receiving antihypertensive treatment, not on a confirmed diagnosis. This distinction is important and should be clarified. Furthermore, Table 1 does not provide sufficient data to verify the presence of hypertension, with only five patient visits meeting the diagnostic definition.***

We thank the reviewer for their comment, as well as their encouragement in their general assessment. We have adapted the language used in the manuscript to show that we are describing the dosing of antihypertensive agents but cannot confirm they were used solely for hypertension. In addition, this is described more explicitly in the Methods section and Limitations subsection of the manuscript. We reproduce both sections below for the reviewer’s convenience:

Methods: “We used receipt of an antihypertensive medication as a proxy for receipt of treatment for hypertension. A previous study of a patient population born <= 32 weeks gestational age and <= 1500 g with no congenital anomalies found that receipt of an antihypertensive medication had a positive predictive value of 75% for having a mention of hypertension in the medical record.[24]”

Limitations: “In addition, the treatment of hypertension was defined in this study using the proxy of receiving any of 14 antihypertensive agents, possibly resulting in case misclassification because some agents may be used for indications other than hypertension. For example, propranolol is commonly used in infants for hemangiomas. We attempted to mitigate this misclassification by excluding participants with a discharge diagnosis of hemangioma in any code position. Additionally, a prior study of over 100,000 NICU admissions found that receipt of any of these medications had a positive predictive value of 75% for a diagnosis of hypertension.[24]”

***3. Presentation of Table 1*
*The current presentation of Table 1 is episode-based, which may obscure important patient-level information. To enhance clarity and provide a more accurate understanding of the cohort, I recommend restructuring Table 1 to present data at the patient level. This would allow for a clearer depiction of comorbidities, particularly when multiple conditions are present in the same individual.***

We thank the reviewer for this suggestion, and we have made the recommended change to Table 1 (as well as the new Supplemental Table 2).

***4. Statistical Support for Group Comparisons*
*Several statements in the manuscript suggest differences between groups without providing statistical evidence. These assertions should either be supported by appropriate statistical tests or rephrased to avoid implying significance where none has been demonstrated.***

We thank the reviewer for their insightful comment. We admit that we have debated about whether to include null hypothesis significance tests of whether individual characteristics vary across the final clusters. The reason we have debated whether to use significance tests is the very reason the reviewer suggests: the group sizes are so small that we would not want readers to conclude that a lack of significance indicates there is no difference among the groups. As the reviewer certainly knows, a lack of significance simply indicates an inconclusive test, not that there are no differences between the clusters. A formal hypothesis test of equivalence is required for such a conclusion.

However, because multiple reviewers have raised this concern, we have decided to include null hypothesis significance tests when comparing variables among the clusters. We will of course include a disclaimer that a lack of significance does not mean that these characteristics *do not* vary across the clusters, simply that we had enough sample size to detect only large differences among the clusters. Future research and larger studies will still be needed.

We have added additional text to this effect at the end of the results section, and we reproduce it below for the reviewer’s convenience:

“While we have included null hypothesis significance tests for among cluster comparisons, many of which were not statistically significant, we remind the reader that the appropriate interpretation of a non-significant hypothesis test is that the differences were “inconclusive.” To conclude that there were “no differences” among clusters, we would have had to conduct a formal hypothesis test of equivalence. The small sample size of some of our clusters led to null hypothesis significance tests that could neither confirm nor disconfirm group differences.”

***5. Development of Cluster Analysis*
*The cluster analysis section would benefit from further elaboration. Incorporating ad hoc statistical tests (e.g. v-test) could enhance the interpretability and robustness of the findings, allowing for more meaningful insights to be drawn from the data.***

We thank the reviewer for the comment. Please see our response to the previous comment directly above this one.

***6. Minor Comments*
*Consider improving the overall readability of the tables by simplifying formatting and ensuring consistency in terminology (e.g. yes/no/missing data)***

We thank the reviewer for their comment and will adjust the formatting of the tables.

***7. Recommendation*
*I encourage the authors to consider these points in a revised version of the manuscript.***

We thank the reviewer for their encouragement. We have considered and addressed each of the critiques, and we believe the changes have significantly improved the paper.
