# Supplementary material for: Dosing trajectories of antihypertensive agents among preterm neonates: A retrospective, cross-sectional analysis
Source: PLoS One. 2025 Dec 8;20(12):e0336994. doi: 10.1371/journal.pone.0336994 (PMC12685197; doi:10.1371/journal.pone.0336994)
Supplement: Attachment — Submitted filename: Response_to_Reviewers_auresp_2.docx [file pone.0336994.s004.docx]

**Reviewer #1: I appreciate the revisions; the authors have fully addressed my comments. I hope this study will serve as a valuable reference for the care of preterm neonates.**

We thank the reviewer for their encouragement, and we are glad that our changes fully addressed the suggestions. The paper is much better now.

**Reviewer #4: The authors have done a great job of taking the reviewers' comments into account, which is commendable. For my part, a minor revision should be done in the conclusion. The authors should be more cautious with the assertion “with hypertension” and replace it with “with probable hypertension.”**

We thank the reviewer for their encouragement. We have changed the two instances in the conclusion from “hypertension” to “probable hypertension”. The new Conclusions section is reproduced below for the reviewer’s convenience:

*“This study provides an improved understanding of the pharmacotherapeutic strategies routinely used in preterm infants with probable hypertension. It specifically provided additional information regarding the doses of medications being used and the timing for when treatment decisions were made. The first step in determining the optimal dosing of agents is to understand what doses are currently being used. Therefore, the results of this study can be used to determine which agents and dosing strategies would have sufficient sample sizes to be compared in observational comparative effectiveness studies. Further multicenter studies evaluating the dosing strategies used by other providers are needed to identify the optimal therapeutic strategies for preterm infants with probable hypertension.”*
